# NONO promotes hepatocellular carcinoma progression by enhancing fatty acids biosynthesis through interacting with ACLY mRNA

**DOI:** 10.1186/s12935-020-01520-4

**Published:** 2020-08-31

**Authors:** Hongda Ding, Junpeng Liu, Caibin Wang, Yang Su

**Affiliations:** Department of General Surgery, Shengjing Hospital of China Medical University, No. 36 Sanhao Road, Shenyang, 110004 China

**Keywords:** Hepatocellular carcinoma, Non-POU domain-containing octamer binding protein, Fatty acid biosynthesis, ATP-citrate lyase, Insulin like growth factor 2 mRNA binding protein 1

## Abstract

**Background:**

Dysregulation of fatty acid (FA) metabolism is involved in hepatocellular carcinoma (HCC) development. Non-POU domain-containing octamer binding protein (NONO), known as the component of nuclear paraspeckles, has recently been found to promote HCC progression. In this study, we investigated the functions of NONO in regulating de novo FA synthesis and its underling mechanism during HCC development.

**Methods:**

The roles of NONO in HCC development by applying gene function loss analysis in HCC cells were detected by quantitative real-time polymerase chain reaction, cell proliferation, and cell invasion assays. The underlying mechanism of NONO in HCC development was examined by western blotting, subcellular fractionation, RNA-binding protein immunoprecipitation-sequencing, chromatin immunoprecipitation, co-immunoprecipitation and mass spectrometry. The effect of NONO on tumorigenesis in vivo was performed with a subcutaneous xenograft mouse model of HCC.

**Results:**

NONO promotes HCC progression by interacting with and increasing ATP-citrate lyase (ACLY) mRNA to enhance FA biosynthesis. Furthermore, NONO promotes ACLY expression through enhancing nuclear ACLY mRNA stability in Diethylnitrosamine-stimulated HCC cells, not related to nuclear paraspeckles. Moreover, we find that NONO/SFPQ (Splicing factor proline and glutamine rich) heterodimer is essential for NONO interacting with ACLY mRNA in DEN stimulated HCC cells. In addition, NONO, Insulin like growth factor 2 mRNA binding protein 1 (IGF2BP1) and ACLY expressions contribute HCC development in mice and are related to poor survival.

**Conclusion:**

NONO promotes HCC progression by enhancing FA biosynthesis through interacting with ACLY mRNA and provide a novel potential target for HCC therapy.

## Background

Hepatocellular carcinoma (HCC) is a highly aggressive solid tumor with poor prognosis and high mortality worldwide [1, 2]. It is estimated that over 700,000 deaths caused by HCC every year globally [3]. Although great progress of HCC therapy, 5-year survival of HCC patients after therapy is still only below 30% [4]. Nowadays, emerging studies report that fatty acids (FA) biosynthesis functions essential role in HCC progression [5–8]. Therefore, clarifying the molecular mechanism of lipid biosynthesis in HCC tumorigenicity and heterogeneity and discovering new therapeutic drugs for HCC are greatly urgent.

Metabolism reprogramming, a distinct characterization of cancer cells, supports energy for the growth and proliferation of cancer cells [9]. For example, the well-known “Warburg phenomenon” is that cancer cells metabolize glucose into lactate under aerobic conditions, not utilizing the route of oxidative decarboxylation by the citric acid cycle for energy production [10]. In contrast, normal cells mainly use the manner of oxidative decarboxylation for energy production [10]. Besides, emerging studies report that dysregulated FA biosynthesis (commonly referred to “de novo FA synthesis”) acts an essential pathogenetic role in the development of many cancers [11]. Generally, cancer cells use cytoplasmic acetyl-CoA as substrate for FA synthesis. Citrate, produced during citric acid cycle in mitochondria, is transported from mitochondria into cytoplasm, and then converted by ATP-citrate lyase (ACLY) into acetyl-CoA and oxaloacetate [12]. In the cytoplasm, acetyl-CoA is converted by acetyl-CoA carboxylase (ACC) to malonyl-CoA. Subsequently, malonyl-CoA molecules and acetyl-CoA are condensed by Fatty acid synthase (FASN) to form palmitate (16 carbon unit) to initiate FA synthesis product [13]. Recently, more and more studies have identified that FA synthesis, such as ACLY, ACC, FASN and stearoyl-CoA-desaturase 1 (SCD1), are highly expressed in many cancers, including HCC [14–17]. However, the underlying mechanism in regulating de novo FA synthesis during HCC development is still not clearly understood [18].

RNA binding protein, non-POU domain-containing octamer binding (NONO), a component of nuclear paraspeckles and plays important roles in various biological processes, including transcriptional regulation, RNA splicing, DNA repair [18, 19]. Besides, recent studies report NONO is involved in development of some tumors [20–22]. For example, NONO promotes breast cancer development via splicing cell proliferation-related pre-mRNA [20]. NONO promotes carcinogenesis through oncogenic isoform switch of bridging integrator 1 in HCC [21]. NONO promotes tumorigenesis of esophageal squamous cell carcinoma through inhibiting apoptosis [22]. Recently, NONO is identified to regulate key metabolic genes expression in response to nutrition [23]. In HCC, the functions of NONO in regulating de novo FA synthesis and its underling mechanism remain unclear.

In this study, we investigated the functions of NONO in regulating de novo FA synthesis and its underling mechanism during HCC development in vivo and in vitro. we find that NONO promotes DEN-induced HCC cell growth and invasion. Through RNA-binding protein immunoprecipitation (RIP)-sequencing, we find that NONO promotes HCC progression by interacting with and increasing ACLY mRNA to enhance FA biosynthesis. Furthermore, NONO promotes ACLY expression through enhancing nuclear ACLY mRNA stability in DEN stimulated HCC cells, not related to nuclear paraspeckles. Moreover, NONO/SFPQ (Splicing factor proline and glutamine rich) heterodimer is essential for NONO interacting with ACLY mRNA in DEN stimulated HCC cells. In addition, NONO, IGF2BP1 and ACLY expressions contribute HCC development in mice and are related to poor survival. Overall, our findings firstly report NONO promotes HCC progression by enhancing FA biosynthesis through interacting with ACLY mRNA and provide a new potential target for HCC therapy.

## Materials and methods

### Ethics statement

The study was approved by the Ethics Committee of ShengJing Hospital of China Medical University. All study participants provided written informed consents.

### Collection of specimens

20 samples of primary HCC tissues were obtained from ShengJing Hospital of China Medical University. No patients had received chemotherapy or radiotherapy prior to surgery. HCC and corresponding normal tissue specimens were obtained immediately after surgical resection and stored at − 80 °C for further analysis.

### Cells, siRNAs, reagents and plasmids

The human HCC cell lines, SMMC-7721 and MHCC97h, were purchased from American Type Culture Collection (ATCC), and were cultured as previously described [24]. siRNAs against NONO (si-NONO), si-ACLY, si-NEAT1_2, si-IGF2BP1 and scrambled siRNA (NC) were purchased and synthetized by Shanghai GenePharma Co., Ltd. The The sequences of these siRNAs were listed in Table 1. *N*-Nitrosodiethylamine (DEN) was purchased from Meilunbio (Dalian, China). NONO, NONO truncations, and SFPQ were amplified by PCR primers listed in Table 1, and then cloned into pCMV-Myc vector or pCMV-Flag vector, as previously described [24].

**Table 1:** Primers used in this study (F, forward; R, reverse)

Name	Sequence	
Primers for NONO constructs		
NONO F	5′-GCCATGGAGGCCCGAATTCGGATGCAGAGTAATAAAACTTTTAACT-3′	pCMV-Myc-NONO
NONO R	5′-GGCCGCGGTACCTCGAGTTAGTATCGGCGACGTTTGTTTG-3′	
N terminal deletion of NONO (ΔN) F	5′-GCCATGGAGGCCCGAATTCGGCGTCTTTTTGTGGGAAATCT-3′	pCMV-Myc-NONO ΔN
N terminal deletion of NONO (ΔN) R	5′-GGCCGCGGTACCTCGAGTTAGTATCGGCGACGTTTGTTTG-3′	
C terminal deletion of NONO (ΔC) F	5′-GCCATGGAGGCCCGAATTCGGATGCAGAGTAATAAAACTTTTAACT-3′	pCMV-Myc-NONO ΔC
C terminal deletion of NONO (ΔC) R	5′-GGCCGCGGTACCTCGAGTTAGTATTCCCTTGAATCCTTCC-3′	
DHBS domain of NONO (DHBS) F	5′-GCCATGGAGGCCCGAATTCGGCGTCTTTTTGTGGGAAATC-3′	pCMV-Myc-NONO DHBS
DHBS domain of NONO (DHBS) R	5′-GGCCGCGGTACCTCGAGTTAGTATTCCCTTGAATCCTTCC-3′	
RRM1 deletion of NONO DHBS domain (DHBSΔR1) F	5′-GCCATGGAGGCCCGAATTCGGTCCCTTACAGTTCGAAACCT-3′	pCMV-Myc-NONO DHBSΔR1
RRM1 deletion of NONO DHBS domain (DHBSΔR1) R	5′-GGCCGCGGTACCTCGAGTTAGTATTCCCTTGAATCCTTCC-3′	
Both RRM1 and RRM2 deletion of NONO DHBS domain (DHBSΔR1 + ΔR2) F	5′-GCCATGGAGGCCCGAATTCGGTTAGATGATGAAGAGGGAC-3′	pCMV-Myc-NONO DHBSΔR1 + ΔR2
Both RRM1 and RRM2 deletion of NONO DHBS domain (DHBSΔR1 + ΔR2) R	5′-GGCCGCGGTACCTCGAGTTAGTATTCCCTTGAATCCTTCC-3′	
SFPQ F	5′-CGGTCGACCATGTCTCGGGATCGGTTC-3′	pCMV-Flag-SFPQ
SFPQ R	5′-CGGGGTACCCTAAAATCGGGGTTTTTT-3′	
Primers for qRT-PCR		
NONO F	5′-CTAGCGGAGATTGCCAAAGTG-3′	
NONO R	5′-GTTCGTTGGACACATACTGAGG-3′	
ACLY F	5′-ATCGGTTCAAGTATGCTCGGG-3′	
ACLY R	5′-GACCAAGTTTTCCACGACGTT-3′	
NEAT1_2 F	5′-CTAGAGGCTCGCATTGTGTG-3′	
NEAT1_2 R	5′-GCCCACACGAAACCTTACAT-3′	
GAPDH F	5′-TCAACAGCAACTCCCACTCTTCCA-3′	
GAPDH R	5′-ACCCTGTTGCTGTAGCCGTATTCA-3′	
The sequences of siRNAs		
si-NONO	5′-CAGGCGAAGUCUUCAUUCA-3′	
si-ACLY	5′-GAUCAAACGUCGUGGAAAAUU-3′	
si-NEAT1_2	5′-GGAGGAGUCAGGAGGAAUAUU-3′	
si-IGF2BP1	5′-CCUGGCUGCUGUAGGUCUU-3′	
Scrambled siRNA	5′-UUCUCCGAACGUGUCACGUTT-3′	
Primers used for ChIP		
ACLY promoter (0–0.5 k) F	5′-GCTGGGATTACAGGCATGAGCCA-3′	
ACLY promoter (0–0.5 k) R	5′-GACTACAGGAGCATGCCACC-3′	
ACLY promoter (0.5–1 k) F	5′-ATAAGATCTAGCCCCAGCTAAGTG-3′	
ACLY promoter (0.5–1 k) R	5′-CAAGAATCGCTTGAACCCGG-3′	
ACLY promoter (1–1.5 k) F	5′-GGCCCGAAGTCCACCGTGCCG-3′	
ACLY promoter (1–1.5 k) R	5′-CGGACCTCACCAAGGCAGGC-3′	
ACLY promoter (1.5–2 k) F	5′-CGGGTTCGGGCCCCGGCTCGG-3′	
ACLY promoter (1.5–2 k) R	5′- CGGGGATCTCTGCAATGGA-3′	

### mRNA stability

To evaluate mRNA stability, MHCC97h cells were stimulated with Actinomycin D (ActD, 10 μg/ml, purchased from Sigma) during indicated times and harvested. Then mRNA expression of ACLY was determined by qRT-PCR.

### Western blotting

SMMC-7721 or MHCC97h cells were treated with DEN (4 mM) or not, lysed, and then subjected to SDS-PAGE and immunoblotting, as previously described [24]. Primary antibodies against NONO (1:500; ab70335, Abcam, USA), ACLY (1:500; ab40793, Abcam, USA), Insulin like growth factor 2 mRNA binding protein 1 (IGF2BP1) (1:500; ab184305, Abcam, USA), Splicing factor proline and glutamine rich (SFPQ) (1:500; ab11825, Abcam, USA), Flag (1:1000; ab1162, Abcam, USA), Myc (1:1000; ab32072, Abcam, USA), and GAPDH (1:1000; ab181602, Abcam, USA) were used.

### RNA isolation and quantitative real-time PCR (qRT-PCR)

SMMC-7721 or MHCC97h Cells were stimulated with DEN (4 mM) or not for the indicated hours, and then harvested. Total RNA was extracted from each sample, using RNA Isolater Total RNA Extraction Reagent (Va-zyme, China). RNA from each sample was reverse-transcribed into cDNA using the PrimeScript RT re-agent kit (Takara, China). qRT-PCR was performed using the 7500 real-time PCR system (Applied Biosystems), with AceQ qPCR SYBR Green Master Mix (Va-zyme, China), as previously described [24]. The obtained data were normalized to GAPDH expression levels in each sample. The primers used in this study were listed in Table 1.

### RNA fractionation

Cellular cytosolic and nuclear RNAs were isolated with a nuclear/cytosol fractionation kit (Biovision, USA), according to the manufacturer’s protocol.

### Transfection

Transfection of plasmids or siRNAs into cells were performed as previously described [24].

### Matrigel invasion assay

Matrigel invasion assay was performed as previously described [24]. Briefly, after matrigel (BD Biosciences, Shanghai, China) was added on the transwell chamber and clotted, cells (10^6^ cells per well) were seeded into the top chamber in 200 μL serum-free media. The bottom well was added with 600 μL complete medium. After 24 h, the matrigel and the cells on the top chamber were removed with cotton swab. The cells on the lower surface of the insert were fixed 4% paraformaldehyde, stained with 0.1% crystal violet and counted from five randomly selected fields and averaged.

### Cell proliferation assay

Cells seeded into a 96-well plate were transfected with siRNAs. After 24 h, the cells were further treated with DEN (4 mM) or not for the indicated time, and then cell proliferation potential was evaluated using MTT Cell Proliferation and Cytotoxicity Assay Kit (Beyotime, China), according to the manufacturer’s instructions, as previously described [24].

### RNA-binding protein immunoprecipitation (RIP) and sequencing

RIP assays were performed essentially as previously described [24]. Briefly, after treatments, cells were harvested and lysed (5 mM HEPES [pH 7.4], 85 mM KCl, 0.5% NP40) for 8 min on ice. After centrifugation, the supernatant was collected and sonicated and 10% of the lysate serves as ‘input’. The remainder of the lysate was incubated with 40 μl protein G-coupled Dynabeads (Life Technologies, USA) for 30 min at 4 °C to decrease the background, followed by washing in lysis buffer and adding protein G-coupled Dynabeads with 3 μg anti-NONO antibody or IgG control, then rotated overnight at 4 °C. RNA was isolated by TRIzol (Invitrogen, USA), incubated with DNase I (Sigma, USA) and reverse-transcribed into cDNA, and subjected to qRT-PCR detection [25]. RNA-seq was performed at Illumina Genome Analyzer II platform at the RiboBio (Guangzhou, China). LifeScope v2.5.1 was used to align the reads to the genome, generate raw counts corresponding to each known gene, and calculate the RPKM (reads per kilobase per million) values. Differentially expressed genes with a fold change > 2 were selected, and gene ontology (GO) analysis was used for pathway enrichment using Cytoscape (ClueGo) with a *P* value < 0.05.

### Chromatin immunoprecipitation (ChIP)

ChIPs were performed using an EZ-Magna ChIP Chromatin Immunoprecipitation Kit (Millipore, USA), as previously described [26, 27]. Briefly, after treatments, cells were crosslinked with 1% formaldehyde for 10 min at room temperature. Then, cell lysis supernatant was carefully removed and nuclear pellets were re-suspended in 0.5 ml of nuclear lysis buffer, then sonicated to create appropriately sized chromatin fragments. After centrifuged to remove insoluble materials, supernatant was transferred to clean microfuge tubes in 50 μl aliquots. Then 3 μg anti-RNA polymerase II (Pol II) antibody (ab264350, Abcam, USA), or normal IgG was added to each nuclear extract, and extracts were further incubated at 4◦C overnight. Nuclear extracts were later incubated with magnetic protein A/G beads for 2 h at 4◦C to capture protein/DNA complexes, then beads were sequentially washed with low salt buffer, high salt buffer, LiCl wash buffer and TE buffer, then protein/DNA complexes were eluted and reverse cross-linked to free the DNA. Purified DNA was analyzed by qPCR with primers listed in Table 1.

### Stable-isotope carbon labeling is traced for flux analysis

MHCC97H cells were cultured in DMEM/F12 medium (17.5 mM unlabeled glucose) supplemented with 7.5 mM [U13C6]-glucose (Cambridge Isotope Laboratories) for 48 h, and total ion chromatography of fatty acids was performed by stable isotope tracing using [U^13^C_6_]-glucose for 48 h. Three independent replicates of 2 × 10^6^ cells for each cell line were collected; the cell pellets were suspended in 0.5 ml of water and lysed by sonication. Cell debris was separated by centrifugation, and proteins were precipitated by treating the clarified supernatant with 1 ml of cold acetone. The final supernatant was air-dried and the free glutamic acid was converted to its trifluoroacetamide butyl ester for GC–MS analysis, as previous described [27, 28].

### Co-immunoprecipitation (Co-IP) and mass spectrometry

Co-IP was performed as previously described [29]. MHCC97H cells in each dish were washed twice with cold phosphate-buffered saline (PBS), collected by scraping, and lysed with 1 ml of modified RIPA buffer (Cell Signaling Technology, Danvers, MA, USA) containing protease and phosphatase inhibitor cocktail (Sigma-Aldrich, St. Louis, MO, USA) for 30 min. Clear lysates were pre-cleared by the addition of 50 μl of protein G bead slurry and incubated at 4 °C overnight with rotation. Supernatants were transferred to a new Eppendorf tube and incubated with 3 μg of anti-NONO antibody or IgG control. with rotation overnight in a cold room; this step was followed by an additional incubation for 3–4 h with protein G beads. The beads were washed three times with RIPA buffer and then boiled in 2× SDS protein loading buffer for 5 min. The purified protein complex was resolved on SDS-PAGE and Coomassie brilliant blue stained. The gel bands of interest were excised from the gel, and proteins specially interacting with NONO were identified by reverse-phase liquid chromatography coupled with tandem mass spectrometry in Beijing Protein Innovation with two replicates. In short, the first dimension reverse-phase separation by micro-LC by a BEH RP C18 column (5 μm, 300 Å, 250 mm × 4.6 mm i.d., Waters Corporation, USA). Mobile phases A (2% acetonitrile, adjusted pH to 10.0 using NH_3_·H_2_O) and B (98% acetonitrile, adjusted pH to 10.0 using NH_3_·H_2_O) were used to develop a gradient. The solvent gradient was set as follows: 5–8% B, 2 min; 8–18% B, 11 min; 18–32% B, 9 min; 32–95% B, 1 min; 95% B, 1 min; 95–5% B, 2 min. The tryptic peptides were separated at an eluent flow rate of 1.0 ml/min and monitored at 214 nm. The column oven was set as 45 °C. Eluent was collected every 90 s. The samples were dried under vacuum and reconstituted in 15 μl of 0.1% (v/v) FA, 2% (v/v) acetonitrile in water for subsequent analyses. Fractions from the first dimension reverse-phase liquid chromatography were dissolved with loading buffer and then separated by a C18 column (75 μm inner-diameter, 360 μm outer-diameter × 15 cm, 2 μm C18). Mobile phase A consisted of 0.1% formic acid in water solution, and mobile phase B consisted of 0.1% formic acid in 80% acetonitrile solution; a series of adjusted linear gradients according to the hydrophobicity of fractions eluted in 1D LC with a flow rate of 300 nL/min was applied. The MS conditions are as the followings: For Orbitrap Fusion Lumos, the source was operated at 1.9 kV, with no sheath gas flow and with the ion transfer tube at 350 °C. The mass spectrometer was programmed to acquire in a data dependent mode. The survey scan was from m/z 350 to 1500 with resolution 60,000 at m/z 200. The 20 most intense peaks with charge state 2 and above were acquired with collision induced dissociation with normalized collision energy of 30%, one microscan and the intensity threshold was set at 1000. The MS2 spectra were acquired with 15, 000 resolution. Peptides were analyzed by Thermo Scientific Q Exactive mass spectrometer, and peptide sequences and protein identity were determined by matching fragmentation patterns in protein databases using the Mascot software program (Matrix Science, Boston, MA). Obtained data from the anti-NONO group has filtered the control data from the IgG group.The MS/MS spectra from each LC–MS/MS run were searched against the raf1.fasta from UniProt using an in-house Proteome Discoverer (Version PD1.4, Thermo-Fisher Scientific, USA). The false discovery rate (FDR) was also set to 0.01 for protein identifications.

### Immunofluorescence assay and RNA fluorescence in situ hybridization (FISH)

MHCC97H cells were grown on cover slips, fixed with 4% paraformaldehyde for 20 min, incubated with proteinase K and washed with a series of alcohol solutions. Then, the slides were washed and incubated with prehybridization solution (BersinBio, Guangzhou, China) for 30 min at 37  °C. Cy3-labeled NEAT1_2 probes (Sangon Biotech, Shanghai, China) were denatured at 73  °C for 8 min and hybridized to the slides for 24 h at 42 °C. Subsequently, cells were then incubated with anti-NONO antibody (ab70335, Abcam, USA) at a 1:100 dilution overnight at 4 °C, followed by further incubation at room temperature for 1 h with secondary antibody and then labeled DNA with DAPI for 10 min. Images were obtained with a confocal microscope (Olympus, Shinjuku, Japan).

### Determination of citrate and oxaloacetate productions

Citrate and oxaloacetate productions in MHCC97H cells were determined by Citrate Assay Kit (ab83396, Abcam, USA) and Oxaloacetate Assay Kit (ab83428, Abcam, USA), according to the manufacturer’s protocol.

### Generation of Knockout cell line with CRISPR/Cas9

Guide RNA sequences for CRISPR/Cas9 were designed at CRISPR design web site (http://crispr.mit.edu/). Insert oligonucleotides for human SFPQ, NONO and IGF2BP1 gRNAs are TCATCCTCCGTGATATCAGCAGG, CACAGGACGAGGAAATCAAGCGG and AACTTTGTAGGGCGTCTCATTGG, respectively. Generation of Knockout MHCC97H cell line with CRISPR/Cas9 was performed as previously described [24].

### Mouse tumor models

Eighteen 4-week-old male BALB/c nude mice were divided into 3 groups randomly. Each group was composed of 6 mice that were injected with 2 × 10^6^ MHCC97H cells (NC), NONO knockouted-MHCC97H cells (NONO-cas9), or IGF2BP1 knockouted-MHCC97H cells (IGF2BP1-cas9). Five weeks later, all mice were killed and the weight of each tumor was measured. Tumor tissues were integrally stripped out. All animal studies were approved by the Animal Ethics Committee of China Medical University and experiments were conducted according to the National Institutes of Health Guide for the Care and Use of Laboratory Animals.

### Immunohistochemistry

Immunohistochemistry was performed as previously described [24]. Briefly, immunohistochemistry staining was performed on 5-μm sections of paraffin-embedded tissue samples to detect ACLY protein expression level. In brief, the slides were incubated in anti- ACLY (1:500; ab40793, Abcam, USA) antibodies at 4 °C overnight. All slides were independently evaluated by two observers. The score for ACLY staining was based on the integrated staining intensity with the average of six randomly selected microscopic fields.

### Statistical analysis

Data were statistically analyzed and graphed using GraphPad Prism 5 (GraphPad Software, San Diego, CA, USA). All results were presented as mean values ± standard deviations. Statistically significant differences between groups were determined by the Student’s t-test. *p < 0.05.

## Results

### NONO promotes DEN-induced HCC cell growth and invasion, and is associated with FA synthesis signaling

**Fig. 1:**
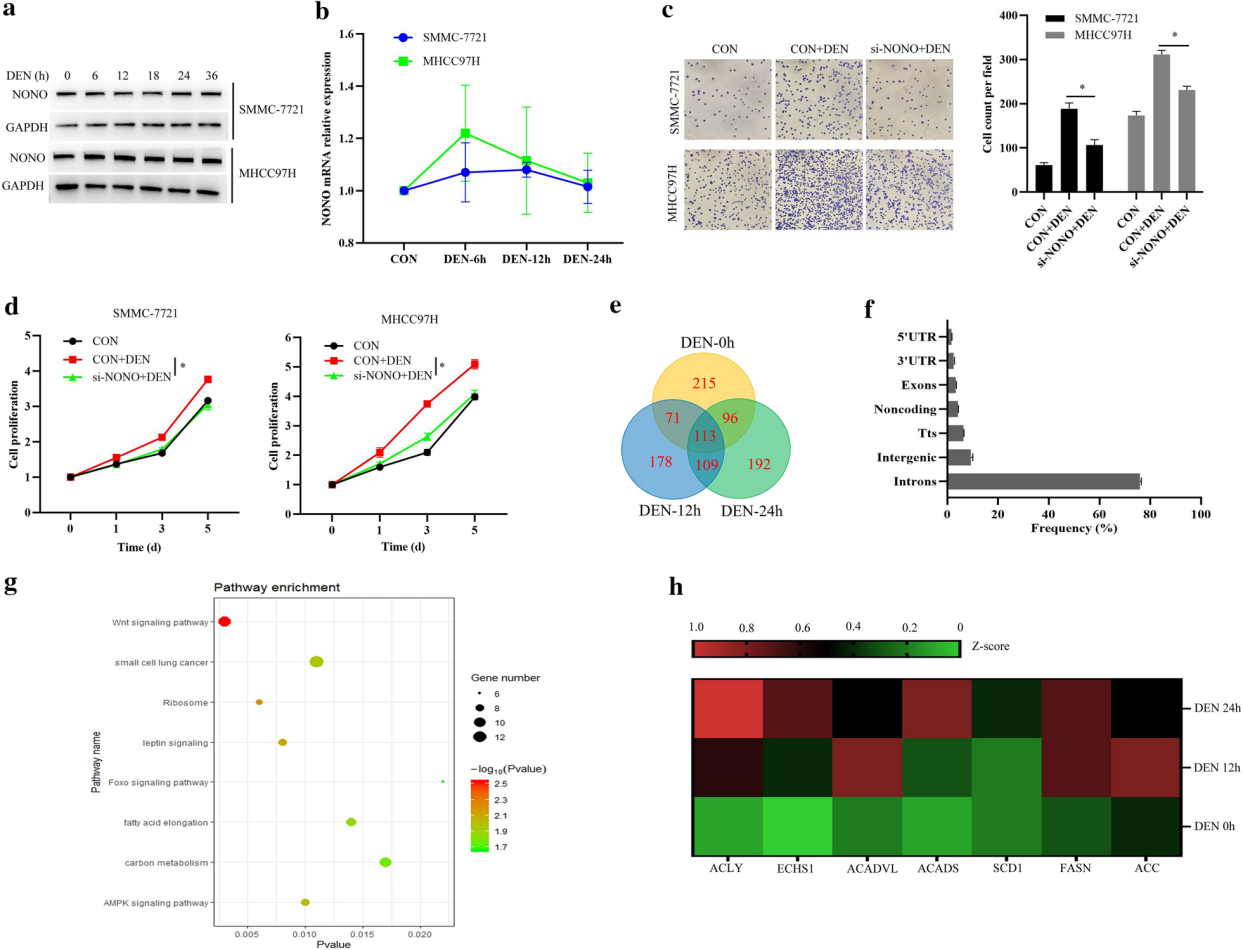
NONO promotes DEN-induced HCC cell growth and invasion, and is associated with FA synthesis signaling. **a** NONO protein expression levels in DEN-stimulated SMMC-7721 or MHCC97H cells were detected by Western blotting. **b** NONO mRNA expression levels in DEN-stimulated SMMC-7721 or MHCC97H cells were detected by qRT-PCR. **c**, **d** SMMC-7721 or MHCC97H cells were transfected with si-NONO for 24 h, and then treated with DEN for 24 h. Cell invasive ability was examined by transwell invasion assays (**c**). Cell proliferation was examined by MTT assays (**d**). **e** Summary of NONO-associated RNAs in DEN-stimulated MHCC97H cells, determined by RIP-seq by precipitation with NONO antibody. **f** Annotation of the NONO RIP-seq peaks. **g** Pathway enrichment analysis of the overlapped 222 genes identified in MHCC97H cells stimulated with DEN for 12 and 24 h. **h** Heatmap analysis of NONO binding genes involved FA biosynthesis. ATP-citrate lyase, ACLY; Short chain enoyl-CoA hydratase 1, ECHS1; Ayl-CoA dehydrogenase very long chain, ACADVL; Acyl-CoA dehydrogenase short chain, ACADS; Stearoyl-CoA-desaturase 1, SCD1; Fatty acid synthase, FASN; Acetyl-CoA carboxylase, ACC. Data are represented as mean ± SD (n = 3; *p < 0.05)

To explore whether NONO is involved in HCC progression, we first detected the expression levels of NONO in HCC cells stimulated with Diethylnitrosamine (DEN), a commonly used drug to induced hepatocarcinogenesis in vivo [30]. As shown in Fig. 1a, b, NONO protein and mRNA expression levels remained relatively stable, and were not significantly changed in HCC cells, SMMC-7721 and MHCC97H during DEN stimulation. Furthermore, we found that NONO knockdown significantly suppressed DEN-induced HCC cell invasion (Fig. 1c) and proliferation (Fig. 1d), suggesting that NONO promotes DEN-induced HCC cell growth and invasion.

Given that NONO is a RNA binding protein, and functions through binding and affecting RNA [31], we performed RIP-Seq to identify NONO-binding RNAs in MHCC97H cells stimulated with DEN. RIP-seq revealed 974 potential candidate targets of NONO, and 178 and 192 genes interacting with NONO were specifically identified in MHCC97H cells stimulated with DEN for 12 or 24 h, respectively (Fig. 1e). Besides, 113 overlapping genes were identified in MHCC97H cells stimulated with DEN for 0, 12 and 24 h (Fig. 1e). Furthermore, the majority of NONO binding sites are located in introns (Fig. 1f). Gene ontology (GO) analysis showed that the overlapped 222 genes identified in MHCC97H cells stimulated with DEN for 12 and 24 h were involved in carbon metabolism, fatty acid elongation, and AMPK (Adenosine Monophosphate-Activated Protein Kinase) signaling (Fig. 1g). In addition, owing to FA biosynthesis has recently been reported to promote HCC progression [9], we further analyzed the NONO binding genes involved FA biosynthesis in the following studies. Heatmap analysis showed that NONO binding genes involved FA biosynthesis, including ACLY, short chain enoyl-CoA hydratase 1 (ECHS1), acyl-CoA dehydrogenase very long chain (ACADVL), acyl-CoA dehydrogenase short chain (ACADS), SCD1, FASN and ACC, were significantly upregulated in HCC cells under DEN stimulation, and ACLY mRNA expression was the most upregulated (Fig. 1h). Overall, these results indicate that NONO promotes DEN-induced HCC cell growth and invasion, and is associated with FA synthesis signaling.

### NONO promotes HCC progression by interacting with ACLY mRNA to enhance FA biosynthesis

**Fig. 2:**
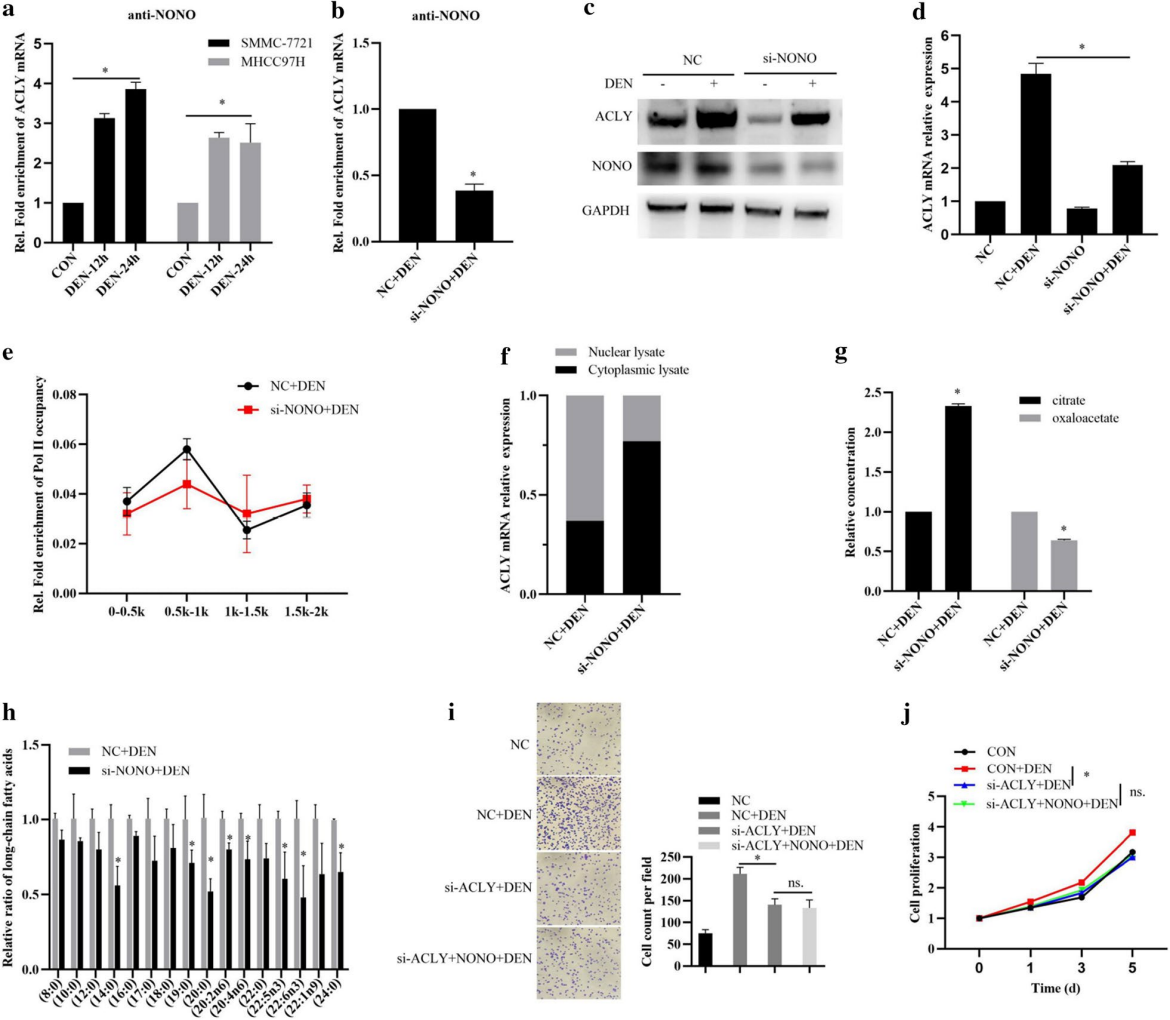
NONO promotes HCC progression by interacting with ACLY mRNA to enhance FA biosynthesis. **a** RIP analysis of the interactions between NONO and ACLY mRNA in DEN-stimulated SMMC-7721 or MHCC97H cells. **b** RIP analysis of the interactions between NONO and ACLY mRNA in MHCC97H cells transfected with si-NONO for 24 h, and then stimulated with DEN for 24 h. **c** Western blotting analysis of the NONO and ACLY protein expressions in MHCC97H cells transfected with si-NONO for 24 h, and then stimulated with DEN for 24 h. **d** qRT-PCR analysis of ACLY mRNA expression in MHCC97H cells transfected with si-NONO for 24 h, and then stimulated with DEN for 24 h. **e** CHIP analysis of the RNA polymerase II (Pol II) binding on ACLY promoter region in MHCC97H cells transfected with si-NONO for 24 h, and then stimulated with DEN for 24 h. **f** qRT-PCR analysis of the distribution ratio of ACLY mRNA in MHCC97H cells transfected with si-NONO for 24 h, and then stimulated with DEN for 24 h. **g** Citrate and oxaloacetate productions were determined in MHCC97H cells transfected with si-NONO for 24 h, and then stimulated with DEN for 24 h. **h** Metabolomics analysis of unsaturated long-chain or polyunsaturated fatty acids in MHCC97H cells transfected with si-NONO for 24 h, and then stimulated with DEN for 24 h. **i**, **j** MHCC97H cells were transfected with si-NONO or/and pCMV-Myc-NONO vector for 24 h, and then treated with DEN for 24 h. Cell invasive ability was examined by transwell invasion assays (**i**). Cell proliferation was examined by MTT assays (**j**). Data are represented as mean ± SD (n = 3; *p < 0.05)

To clarify whether NONO promoting HCC progression is related to FA biosynthesis, we next examined the interactions between NONO and ACLY mRNA by RIP. The results showed that DEN stimulation significantly enhanced NONO interacting with ACLY mRNA both in SMMC-7721 and MHCC97H cells (Fig. 2a). Furthermore, DEN stimulation evidently promoted ACLY mRNA and protein expression in HCC cells, and NONO knockdown significantly inhibited the increase of ACLY mRNA and protein expression (Fig. 2b–d). Whereas, chromatin immunoprecipitation (ChIP) analysis showed that NONO knockdown did not significantly affect RNA polymerase II (Pol II), which is the sole enzyme responsible for gene transcription [32], binding to ACLY promoter region (Fig. 2e). Then, we detected the distribution ratio of ACLY mRNA and found that NONO knockdown increased the cytoplasmic distribution of ACLY mRNA, but decreased nuclear distribution of ACLY mRNA, suggesting NONO may affect ACLY mRNA transport from nucleus to cytoplasm (Fig. 2f).

Since ACLY is responsible for converting citrate into acetyl-CoA and oxaloacetate during the process of FA biosynthesis [12], we then further investigated whether NONO affected oxaloacetate production in DEN-stimulated HCC cells. As shown in Fig. 2g, NONO knockdown significantly decreased citrate production, and increased oxaloacetate production. Next, the effects of NONO on FA biosynthesis in DEN-stimulated HCC cells by examining the metabolization of free fatty acids of varying carbon lengths and C–C bond unsaturation through metabolomic analysis. The results showed that NONO knockdown evidently suppressed fatty acid elongation in DEN-stimulated HCC cells (Fig. 2h). Moreover, ACLY knockdown significantly inhibited DEN-induced HCC cell growth and invasion, whereas NONO overexpression did not reversed the inhibitory effects of ACLY knockdown on DEN-induced HCC cell growth and invasion (Fig. 2i, j), indicating that NONO promoting HCC progression depends on ACLY. Taken together, these results suggest that NONO promotes HCC progression by interacting with ACLY mRNA to enhance FA biosynthesis.

### NONO promoting ACLY expression is not related to nuclear paraspeckles in HCC cells

**Fig. 3:**
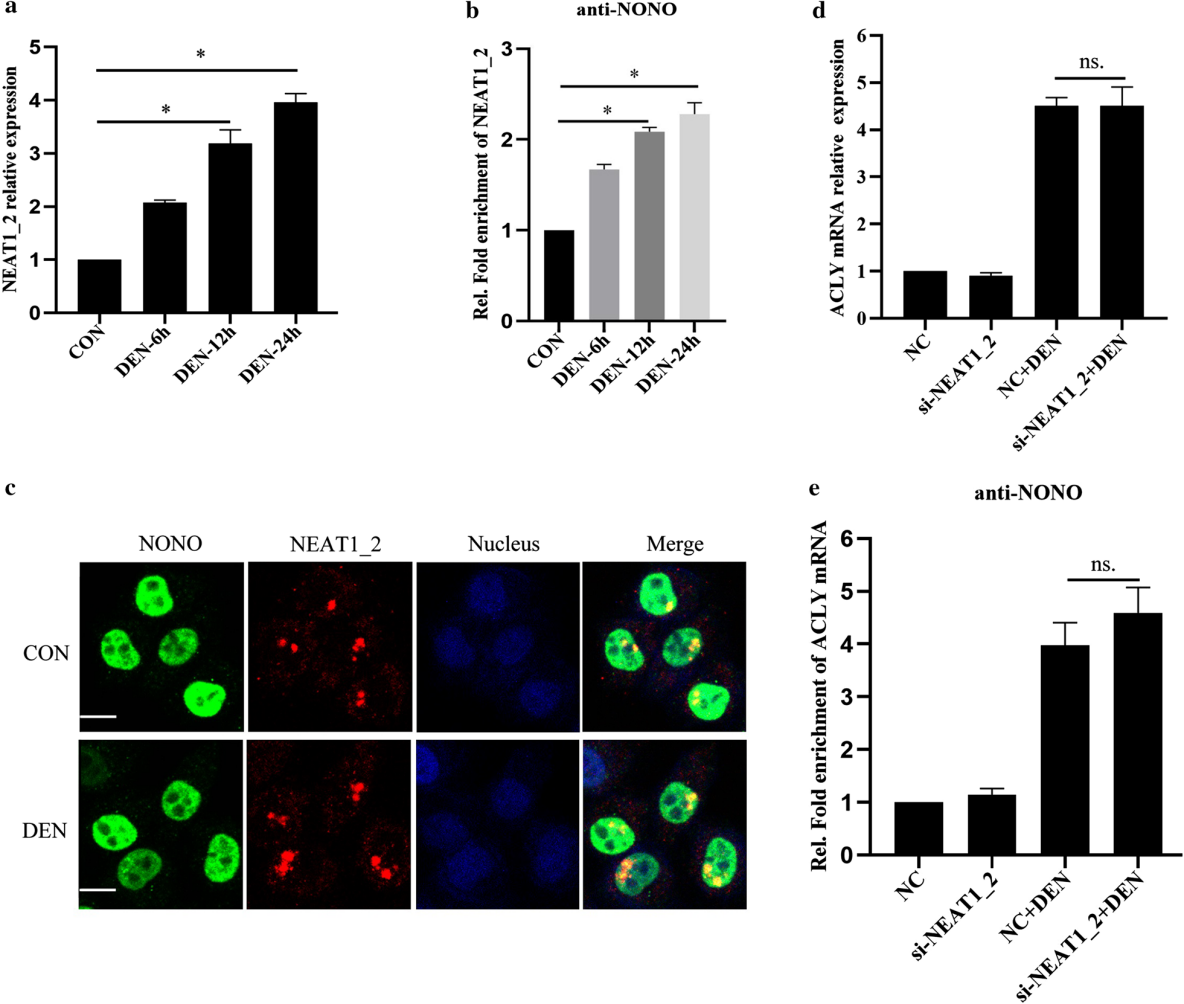
NONO promoting ACLY expression is not related to nuclear paraspeckles in HCC cells. **a** NEAT1_2 expressions in DEN-stimulated MHCC97H cells were detected by qRT-PCR. **b** RIP analysis of the interactions between NONO and NEAT1_2 in DEN-stimulated MHCC97H cells. **c** Immunofluorescence analysis of NONO and NEAT1_2 distributions in DEN-stimulated MHCC97H cells (NONO, green; NEAT1_2, representative of Paraspeckle, red; Nucleus, blue). **d** qRT-PCR analysis of ACLY mRNA expression in MHCC97H cells transfected with si-NEAT1_2 for 24 h, and then stimulated with DEN for 24 h. **e** RIP analysis of the interactions between NONO and ACLY mRNA in MHCC97H cells transfected with si-NEAT1_2 for 24 h, and then stimulated with DEN for 24 h. Data are represented as mean ± SD (n = 3; *p < 0.05)

Given NONO is an important component of nuclear paraspeckles, which have been demonstrated to promote HCC progression and induce HCC chemoresistance [33, 34], we further investigate NONO promoting ACLY expression in DEN-stimulated HCC cells is associated with nuclear paraspeckle. As shown in Fig. 3a, DEN stimulation significantly increased nuclear paraspeckle assembly transcript 1_2 (NEAT1_2) expression in HCC cells, which is essential for de novo paraspeckle assembly [35]. Furthermore, RIP analysis showed that DEN stimulation significantly promoted NONO binding to NEAT1_2 in HCC cells (Fig. 3b). Consistently, DEN stimulation evidently increased paraspeckles in HCC cells, analyzed by RNA immunofluorescence (Fig. 3c). Moreover, NEAT1_2 knockdown neither affected upregulated expression of ACLY mRNA in HCC cells stimulated with DEN (Fig. 3d), nor inhibited NONO interacting with ACLY mRNA (Fig. 3e). Overall, these results indicate that NONO promoting ACLY expression is not related to nuclear paraspeckles in HCC cells.

### NONO promotes nuclear ACLY mRNA stability, and IGF2BP1 increases cytoplastic ACLY mRNA stability in DEN stimulated HCC cells

**Fig. 4:**
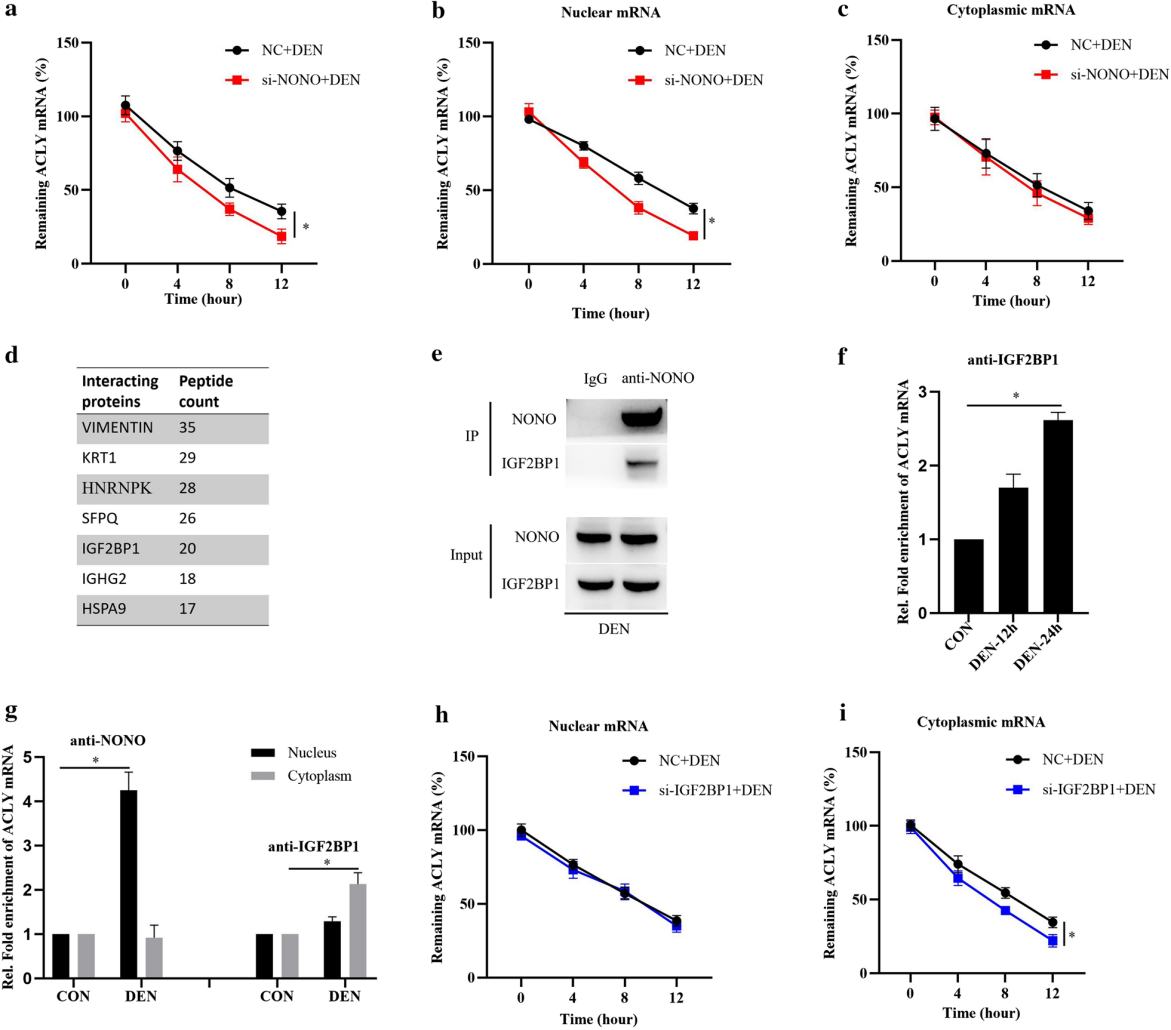
NONO promotes nuclear ACLY mRNA stability, and IGF2BP1 increases cytoplastic ACLY mRNA stability in DEN stimulated HCC cells. **a**–**c** qRT-PCR analysis of whole ACLY mRNA expression (**a**), nuclear ACLY mRNA expression (**b**), or cytoplasmic ACLY mRNA expression (**c**) in MHCC97H cells transfected with si-NONO for 24 h, stimulated with DEN for 24 h, then treated with the transcriptional inhibitor Actinomycin D (ActD, 10 μg/ml) for indicated hours. **d** Proteins interacted with NONO in MHCC97H cells identified with COIP. **e** COIP analysis of the interactions of NONO and IGF2BP1 in DEN-stimulated MHCC97H cells. **f** RIP analysis of the interactions between IGF2BP1 and ACLY mRNA in DEN-stimulated MHCC97H cells. **g** RIP analysis of the interactions between NONO/IGF2BP1 and ACLY mRNA in the nuclear or cytoplasmic fractions of DEN-stimulated MHCC97H cells. **h**, **i** qRT-PCR analysis of nuclear ACLY mRNA expression (**h**), or cytoplasmic ACLY mRNA expression (**i**) in MHCC97H cells transfected with si-IGF2BP1 for 24 h, stimulated with DEN for 24 h, then treated with ActD for indicated hours. Data are represented as mean ± SD (n = 3; *p < 0.05)

To further elucidate the underlying mechanism on NONO promoting ACLY mRNA expression in HCC, we explored the effects of NONO on ACLY mRNA stability in HCC cells. As shown in Fig. 4a, NONO knockdown significantly decreased the half-life time of ACLY mRNA in DEN stimulated HCC cells. Unexpected, NONO knockdown only affected the half-life time of nuclear ACLY mRNA (Fig. 4b), but not cytoplasmic ACLY mRNA (Fig. 4c). Subsequently, we investigated the underlying molecular mechanism on NONO increasing ACLY mRNA stability by co-IP assay using specific anti-NONO antibody accompanied with mass spectrometry to identify the proteins interacting with NONO in HCC cells. Among the identified proteins interacting with NONO, we focused on insulin like growth factor 2 mRNA binding protein 1 (IGF2BP1) (Fig. 4d), which has been shown to promote mRNAs stability in the cytoplasm via binding m^6^A mRNAs [36]. Indeed, endogenous co-IP analysis confirmed NONO interacted with IGF2BP1 in HCC cells (Fig. 4e). Furthermore, we found that DEN stimulation also significantly promoted IGF2BP1 binding to ACLY mRNA (Fig. 4f). Moreover, DEN stimulation increased IGF2BP1 binding to cytoplasmic ACLY mRNA, whereas DEN stimulation promoted NONO binding to nuclear ACLY mRNA (Fig. 4g). In addition, IGF2BP1 knockdown evidently decreased the half-life time of ACLY mRNA in the cytoplasm (Fig. 4i), but not in the nucleus (Fig. 4h). Taken together, these results suggest that NONO promotes nuclear ACLY mRNA stability, and IGF2BP1 increases cytoplastic ACLY mRNA stability in DEN stimulated HCC cells.

### NONO/SFPQ heterodimer is essential for NONO interacting with ACLY mRNA in HCC cells

**Fig. 5:**
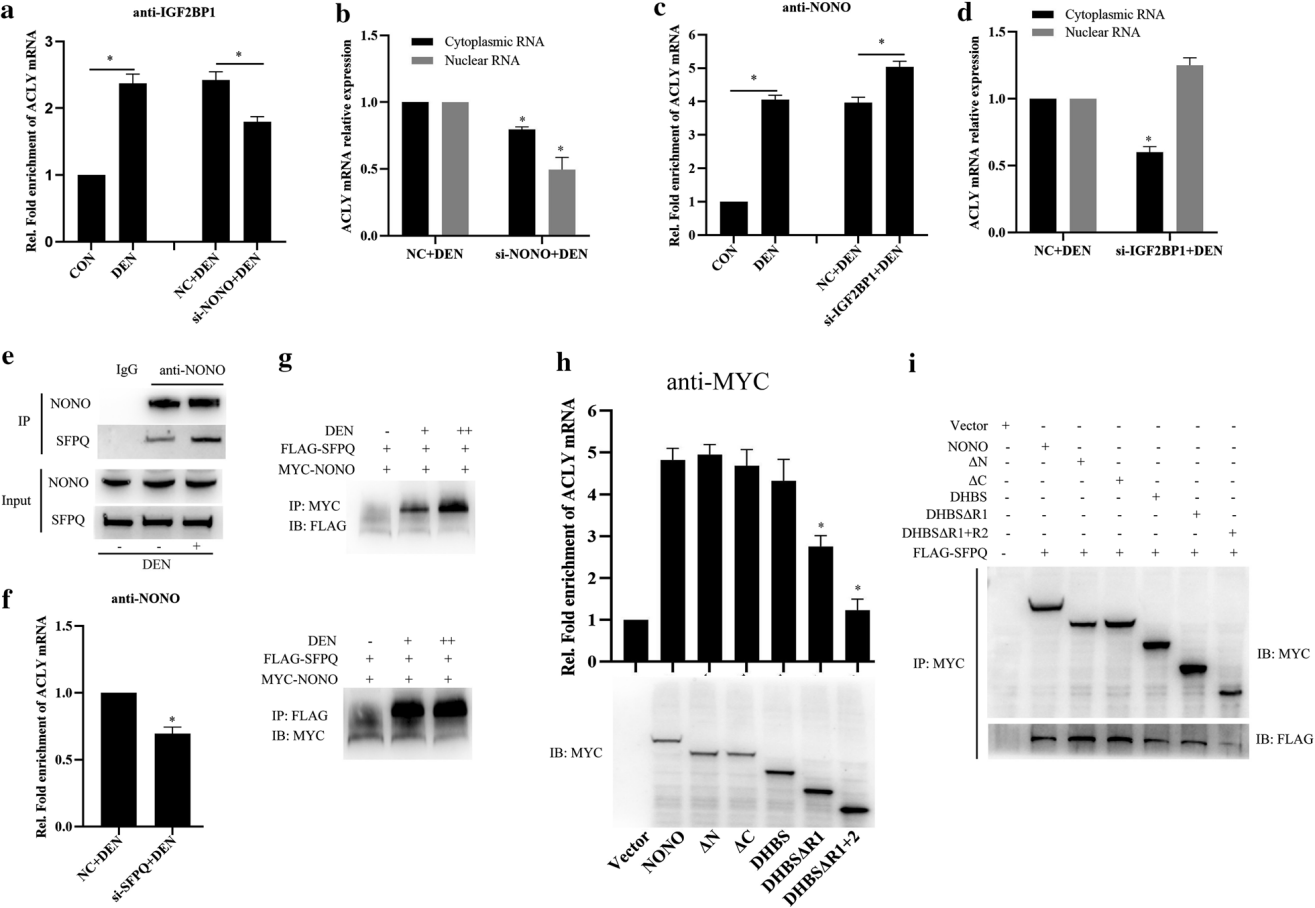
NONO and SFPQ promotes ACLY mRNA stability in HCC cells. **a** RIP analysis of the interactions between IGF2BP1 and ACLY mRNA in MHCC97H cells transfected with si-NONO for 24 h, and then stimulated with DEN for 24 h. **b** qRT-PCR analysis of nuclear and cytoplasmic ACLY mRNA expression in MHCC97H cells transfected with si-NONO for 24 h, and then stimulated with DEN for 24 h. **c** RIP analysis of the interactions between NONO and ACLY mRNA in MHCC97H cells transfected with si-IGF2BP1 for 24 h, and then stimulated with DEN for 24 h. **d** qRT-PCR analysis of nuclear and cytoplasmic ACLY mRNA expression in MHCC97H cells transfected with si-IGF2BP1 for 24 h, and then stimulated with DEN for 24 h. **e** COIP analysis of the interactions of NONO and SFPQ in DEN-stimulated MHCC97H cells. **f** RIP analysis of the interactions between NONO and ACLY mRNA in MHCC97H cells transfected with si-SFPQ for 24 h, and then stimulated with DEN for 24 h. **g** COIP analysis of the interactions of MYC-NONO and FLAG-SFPQ in DEN-stimulated MHCC97H cells. **h** RIP analysis of the interactions between MYC-NONO or NONO truncations and ACLY mRNA in MHCC97H cells. **i** COIP analysis of the interacting domain of NONO with FLAG-SFPQ in MHCC97H cells. Data are represented as mean ± SD (n = 3; *p < 0.05)

Subsequently, we further investigated whether NONO promoting ACLY mRNA stability was associated with NONO interacting with IGF2BP1 in DEN stimulated HCC cells. RIP analysis showed that NONO knockdown significantly decreased IGF2BP1 binding to ACLY mRNA in DEN stimulated HCC cells (Fig. 5a). Given that NONO is involved in regulating nuclear retention of mRNAs [37], we further examined the effects of NONO on ACLY mRNA distribution and found that NONO knockdown significantly decreased ACLY mRNA expression both in the cytoplasm and nucleus of DEN stimulated HCC cells (Fig. 5b), which may partially explain why NONO knockdown decreasing IGF2BP1 binding to ACLY mRNA (Fig. 5a). Furthermore, we found that IGF2BP1 knockdown slightly increased NONO binding to ACLY mRNA in DEN stimulated HCC cells (Fig. 5c), and significantly increased ACLY mRNA expression in the nucleus of DEN stimulated HCC cells, but decreased ACLY mRNA expression in the cytoplasm (Fig. 5d), suggesting that NONO functions on the upstream of nucleocytoplasmic export of ACLY mRNA. In addition, NONO knockdown decreases the total ACLY mRNA (Fig. 5b), but IGF2BP1 knockdown seems to disturb the nucleocytoplasmic export of ACLY mRNA, which promotes ACLY mRNA retention in nucleus.

Given that NONO trends to interact with splicing factor proline- and glutamine-rich (SFPQ) to form heterodimer to regulate paraspeckle formation, microRNA synthesis, transcription and so on [38], we then further investigated whether NONO/SFPQ heterodimer affects NONO binding to ACLY mRNA. We found that DEN stimulation significantly promoted NONO interacting with SFPQ and heterodimer formation in HCC cells (Fig. 5e, g) and SFPQ knockdown inhibited NONO binding to ACLY mRNA in DEN stimulated HCC cells (Fig. 5f). In order to clarify the interacting domain, we constructed a series of truncations of NONO and performed RIPs. As shown in Fig. 5h, neither N terminal or C terminal domain deletion affected the binding between NONO and ACLY mRNA, but RNA binding domain RRM1 deletion or both RRM1 and RRM2 deletion decreased the binding between NONO and ACLY mRNA (Fig. 5h). In addition, NONO mutant of RRM1 and RRM2 deletion also affects the association between SFPQ and NONO (Fig. 5I).

**Fig. 6:**
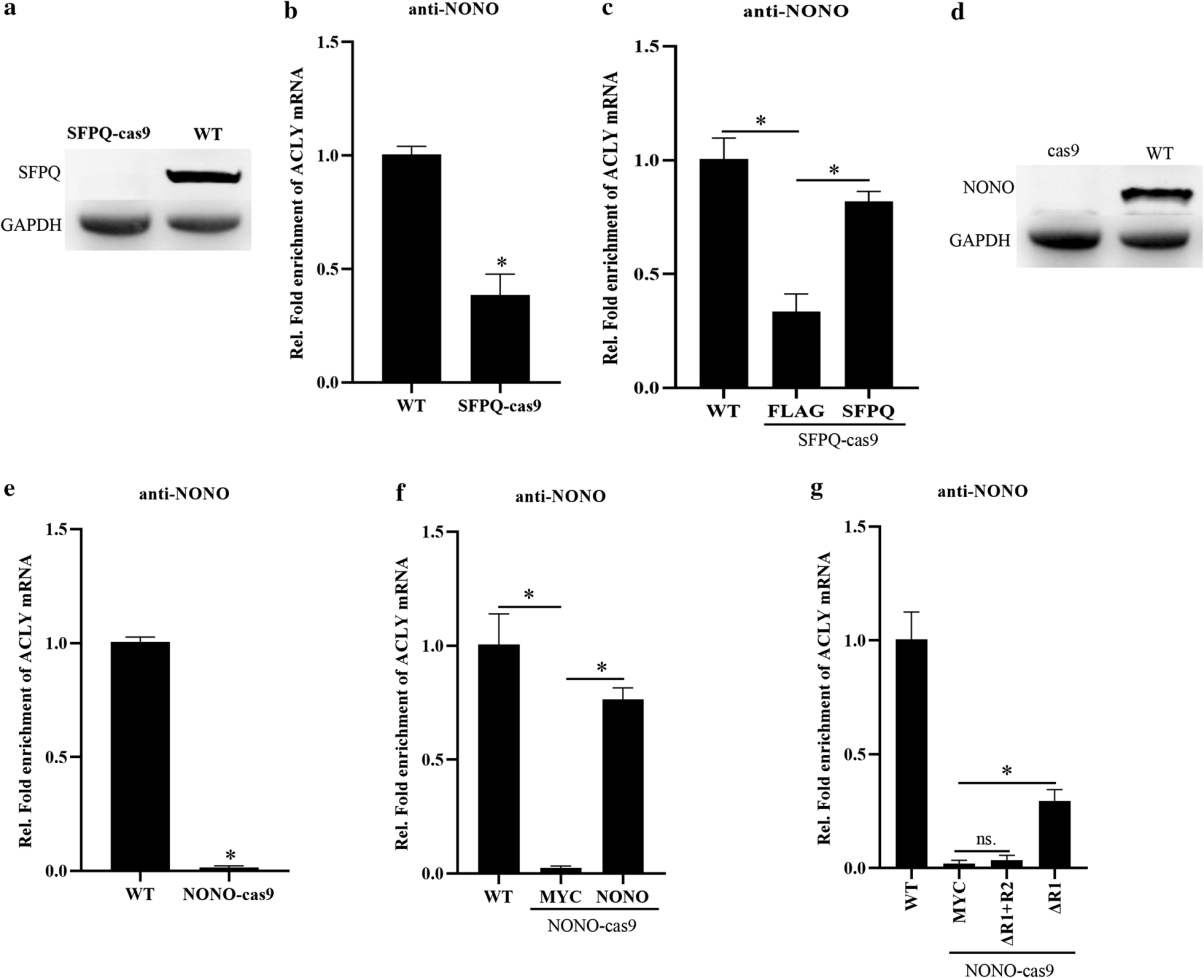
NONO/SFPQ heterodimer is essential for NONO interacting with ACLY mRNA in HCC cells. **a** Western blotting analysis of SFPQ expression in wild type MHCC97H cells (WT) or SFPQ-knockout MHCC97H cells (SFPQ-Cas9). **b** RIP analysis of the interactions between NONO and ACLY mRNA in wild type MHCC97H cells (WT) or SFPQ-knockout MHCC97H cells (SFPQ-Cas9). **c** RIP analysis of the interactions between NONO and ACLY mRNA in wild type MHCC97H cells (WT), SFPQ-knockout MHCC97H cells transfected with pCMV-FLAG vector or pCMV-FLAG-SFPQ. **d** Western blotting analysis of NONO expression in wild type MHCC97H cells (WT) or NONO-knockout MHCC97H cells (NONO-Cas9). **e** RIP analysis of the interactions between NONO and ACLY mRNA in wild type MHCC97H cells (WT) or NONO-knockout MHCC97H cells (NONO-Cas9). **f** RIP analysis of the interactions between NONO and ACLY mRNA in wild type MHCC97H cells (WT), NONO-knockout MHCC97H cells (NONO-Cas9) transfected with pCMV-MYC vector or pCMV-MYC-NONO. **g** RIP analysis of the interactions between NONO and ACLY mRNA in wild type MHCC97H cells (WT), NONO-knockout MHCC97H cells (NONO-Cas9) transfected with pCMV-MYC vector, pCMV-MYC-NONO ΔR1 + R2 or pCMV-MYC-NONO ΔR1. Data are represented as mean ± SD (n = 3; *p < 0.05)

To further explore the role of NONO interacting with SFPQ on NONO binding with ACLY mRNA, we generated a SFPQ knockout MHCC97H cell line by the CRISPR/Cas9 method (Fig. 6a) and found SFPQ deletion decreased the binding of NONO and ACLY mRNA (Fig. 6b), consistent with the result of Fig. 5f. Furthermore, ectopic expression of SFPQ partly rescued the binding of NONO and ACLY mRNA (Fig. 6c). However, NONO deletion completely destroyed the binding of NONO and ACLY mRNA (Fig. 6d, e). Ectopic expression of NONO or mutant of RRM1 deletion, but not mutant of RRM1 and RRM2 deletion, partly rescued the binding of NONO and ACLY mRNA (Fig. 6f, g). Taken together, these results suggest that NONO/SFPQ heterodimer is essential for NONO interacting with ACLY mRNA in HCC cells.

### IGF2BP1, NONO and ACLY expressions contribute HCC development in mice and are related to poor survival

**Fig. 7:**
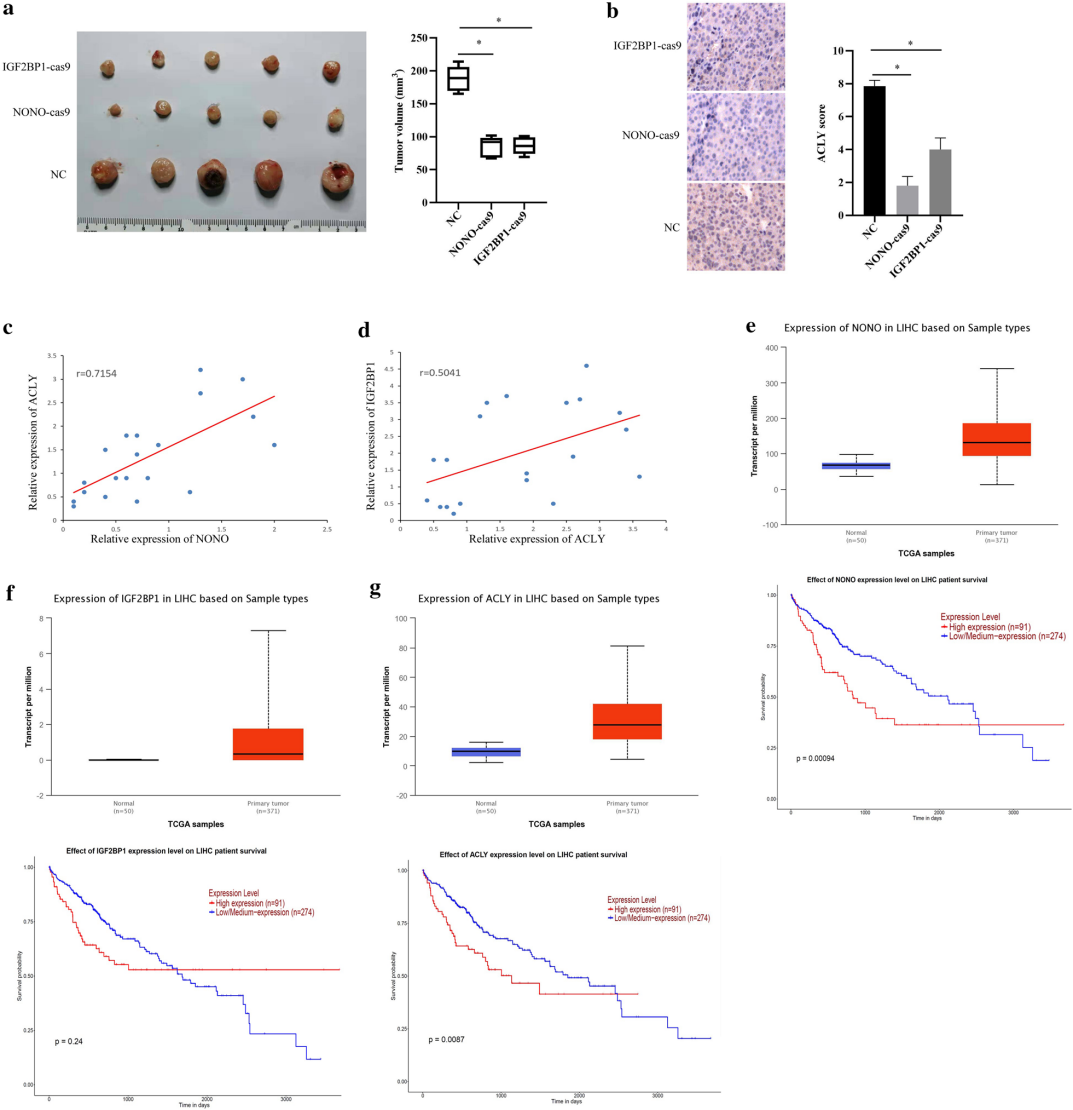
IGF2BP1, NONO and ACLY expressions contribute HCC development in mice and are related to poor survival. **a** IGF2BP1 or NONO knockout significantly inhibits tumor growth in vivo. Representative images of xenograft tumors from the nude mice. **b** Immunochemistry analysis of ACLY in tumor tissues (× 200 magnification). mRNA **c** Correlational analysis of NONO (C)/IGF2BP1 **d** protein and ACLY mRNA expression in clinical HCC tissues. **e** NONO mRNA expression analysis using TCGA database, and survival analysis of high or low expression of NONO on liver hepatocellular carcinoma patient from TCGA database (p = 0.00094). **f** IGF2BP1 mRNA expression analysis using TCGA database, and survival analysis of high or low expression of IGF2BP1 on liver hepatocellular carcinoma patient from TCGA database (p = 0.24). **g** ACLY mRNA expression analysis using TCGA database, and survival analysis of high or low expression of ACLY on liver hepatocellular carcinoma patient from TCGA database (p = 0.0087). Data are represented as mean ± SD (n = 3; *p < 0.05)

To investigate the tumorigenesis effects of IGF2BP1 and NONO in vivo, we subcutaneously injected wild type (WT), IGF2BP1-knockout (IGF2BP1-cas9), or NONO-knockout (NONO-cas9) MHCC97H cells in nude mice and found that IGF2BP1 knockout or NONO knockout caused less tumor formation and evidently reduced tumor size, compared with control group (Fig. 7a). Besides, immunochemistry analysis showed that ACLY expression was evidently decreased in the tumor tissues of IGF2BP1 knockout group or NONO knockout group, compared to that in control group (Fig. 7b). Furthermore, we detected the mRNA expression levels of IGF2BP1, NONO and ACLY in clinical HCC tissue samples, and found that IGF2BP1 mRNA or NONO mRNA expression was positively related to ACLY mRNA expression in a linearly dependent-manner in clinical HCC tissue samples (Fig. 7c, d). Moreover, we analyzed TCGA database and found that IGF2BP1, NONO and ACLY are relatively highly expressed in liver hepatocellular carcinoma, compared to normal tissue (Fig. 6e–g), and highly expressed IGF2BP1, NONO and ACLY are related to the poor survival (Fig. 7e–g). Overall, these results indicate that IGF2BP1 and NONO are frequently upregulated in HCC tissues and promote tumor masses.

## Discussion

HCC is a complex multifactorial tricky problem globally [1]. Deeply exploring the molecular mechanism during HCC development is essential for clinical therapy discovery. NONO, known as the component of nuclear paraspeckles, has been reported to promote breast cancer, esophageal squamous cell, and advanced prostate cancer [20, 22, 39]. Recent study indicates that NONO expression is significantly associated with poor survival of HCC patients [33], whereas the underlying mechanism of NONO in HCC progression is not well known. In this study, our group explored the new role of NONO during DEN-induced HCC development and found the interaction of NONO and IGF2BP1 regulates nuclear and cytoplasmic ACLY mRNA stability, respectively. In nucleus, NONO interacts with SFPQ to bind ACLY mRNA to suppress degradation. And, NONO also transports the nuclear ACLY mRNA to cytoplasm by interacting with IGF2BP1. IGF2BP1 binds to ACLY mRNA to inhibit degradation in cytoplasm and promote fatty acid synthesize. Though we preliminarily clarify the main route of NONO and IGF2BP1 complex regulating ACLY mRNA in HCC cells, there are many details needed to be investigated further. Our study precludes the role of NONO-containing paraspeckle on NONO-mediated ACLY expression, but DEN treatment also induced paraspeckle formation in HCC cells. Given that paraspeckle mainly functions by remaining mRNA or protein [37], it seems that NONO-containing paraspeckle also plays some role during DEN-induced HCC progression which needs to be explored later. Previous study had demonstrated IGF2BP1 regulated target mRNA stability on m6A-dependent manner [36]. In our study, IGF2BP1 also regulates mRNA stability of ACLY. Whether m6A-mediated ACLY mRNA modifications involved in NONO-IGF2BP1 mRNA transport is unknown. While NONO locates in nucleus and IGF2BP1 distributes in cytoplasm and nucleus, but NONO mainly controls ACLY mRNA nuclear stability and IGF2BP1 mainly controls ACLY mRNA cytoplasmic stability, the particular molecular event involved in ACLY mRNA transport from NONO to IGF2BP1 is also unclear.

In most studies, NONO functions as a mRNA retention protein, but NONO of our study functions as a transport-related protein which is contradictory. In our view, NONO binding mRNA is temporary, and these binding mRNAs should be moving and changing. Our study maybe firstly discovering the downstream adaptor protein of NONO retention signaling.

## Conclusion

Our findings firstly report NONO promotes HCC progression by enhancing FA biosynthesis through interacting with ACLY mRNA and provide a novel potential target for HCC therapy.

## Supplementary information

**Additional file 1.** Additional figures.

## Data Availability

The data in this study are available from the author for correspondence upon reasonable request.
