# Supplementary material for: NONO promotes hepatocellular carcinoma progression by enhancing fatty acids biosynthesis through interacting with ACLY mRNA
Source: Cancer Cell Int. 2020 Aug 31;20:425. doi: 10.1186/s12935-020-01520-4 (PMC7461318; doi:10.1186/s12935-020-01520-4)
Supplement: Supplementary file 1 — Additional file 1. Additional figures. [file 12935_2020_1520_MOESM1_ESM.doc]

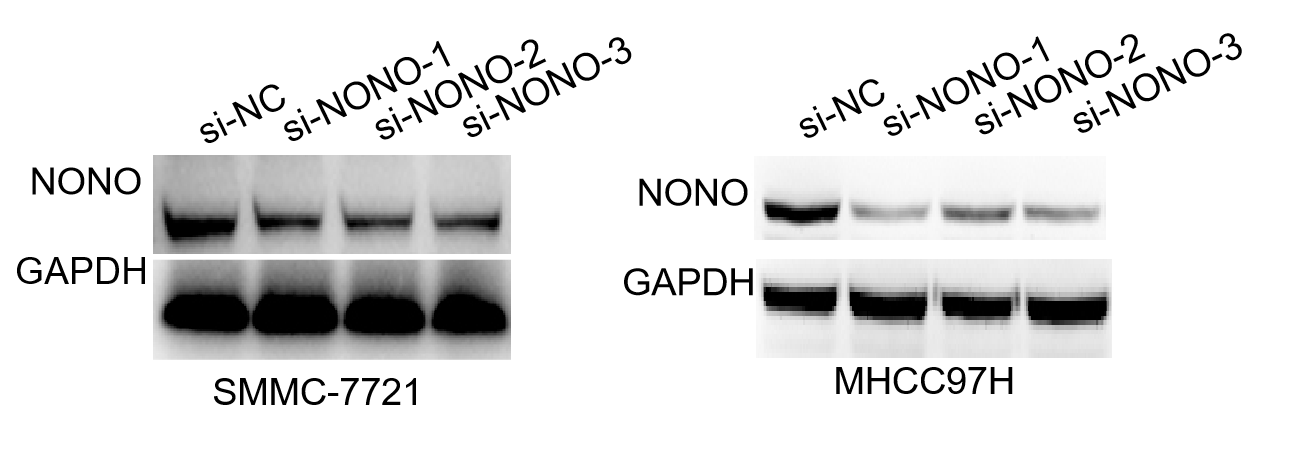


**Additional file 1: Fig. 1.** Western blottig analysis of knockdown effects of siRNA against-NONO in SMMC-7721 or MHCC97H cells.


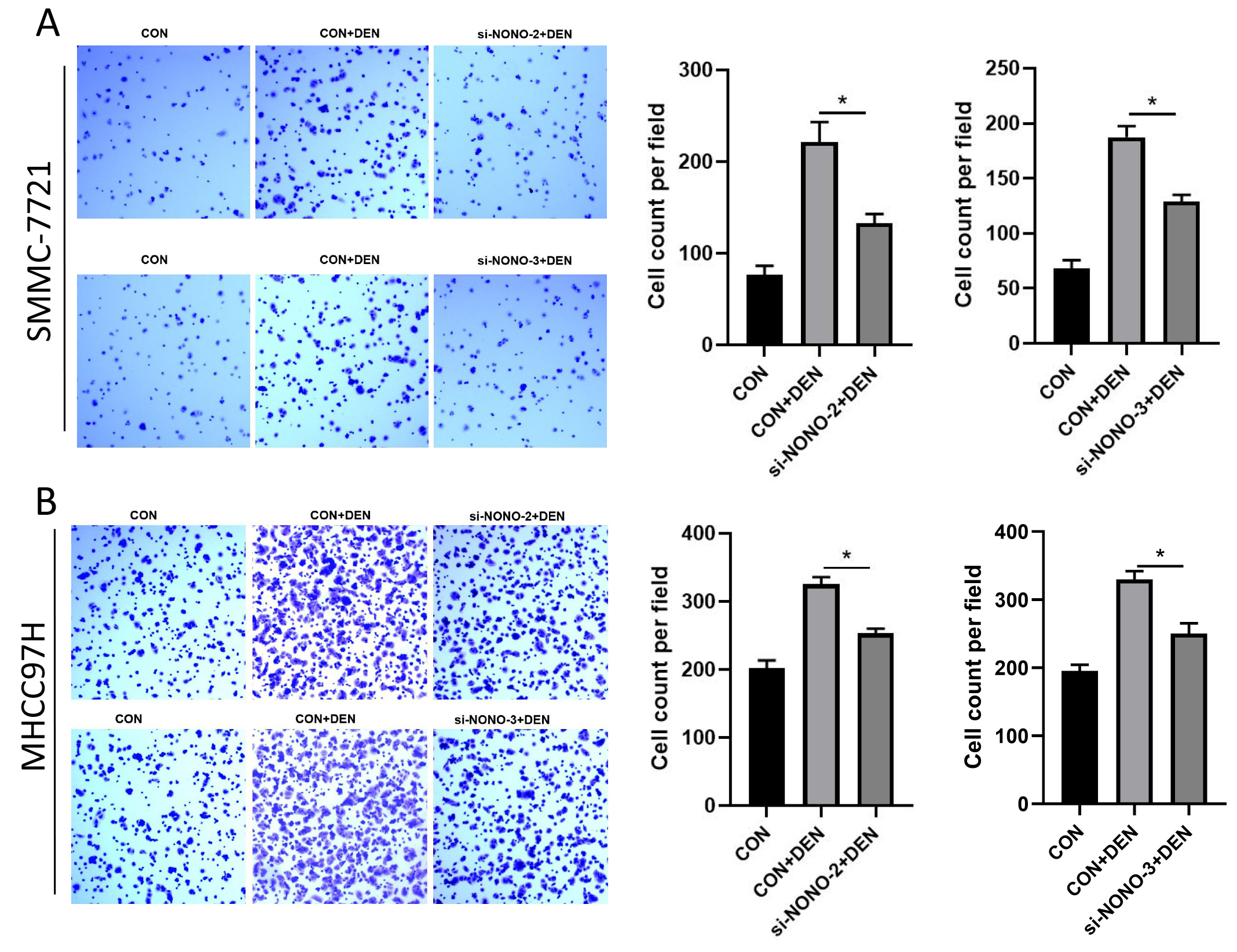


**Additional file 1: Fig. 2.** SMMC-7721 (A) or MHCC97H (B) cells were transfected with si-NONO-2/3 for 24 h, and then treated with DEN for 24 h. Cell invasive ability was examined by transwell invasion assays.
